# Multi-machine learning model based on radiomics features to predict prognosis of muscle-invasive bladder cancer

**DOI:** 10.1186/s12885-025-14279-6

**Published:** 2025-07-01

**Authors:** Bin Wang, Zijian Gong, Peide Su, Guanghao Zhen, Tao Zeng, Yinquan Ye

**Affiliations:** 1 The Second Affiliated Hospital, Jiangxi Medical College, Nanchang University, Nanchang, China; 2 Department of Urology, Second Affiliated Hospital of Nanchang University, Nanchang, China; 3 Department of Radiology, The Second Affiliated Hospital, Jiangxi Medical College, Nanchang University, Nanchang, 330006 China; 4 Intelligent Medical Imaging of Jiangxi Key Laboratory, Nanchang, 330006 China

**Keywords:** CT, Bladder cancer, Machine learning, Radiomics, Prognosis

## Abstract

**Objective:**

This study aims to construct a survival prognosis prediction model for muscle-invasive bladder cancer based on CT imaging features.

**Materials and methods:**

A total of 91 patients with muscle-invasive bladder cancer were sourced from the TCGA and TCIA dataset and were divided into a training group (64 cases) and a validation group (27 cases). Additionally, 54 patients with muscle-invasive bladder cancer were retrospectively collected from our hospital to serve as an external test group; their enhanced CT imaging data were analyzed and processed to identify the most relevant radiomic features. Five distinct machine learning methods were employed to develop the optimal radiomics model, which was then combined with clinical data to create a nomogram model aimed at accurately predicting the overall survival (OS) of patients with muscle-invasive bladder cancer. The model’s performance was ultimately assessed using various evaluation methods, including the ROC curve, calibration curve, decision curve, and Kaplan-Meier (KM) analysis.

**Results:**

Eight radiomic features were identified for modeling analysis. Among the models evaluated, the Gradient Boosting Machine (GBM) In the prediction of OS performed the best. the 2-year AUCs were 0.859, 95% CI (0.767–0.952) for the training group, 0.850, 95% CI (0.705–0.995) for the validation group, and 0.700, 95% CI (0.520–0.880) for the external test group. The 3-year AUCs were 0.809, 95% CI (0.704–0.913) for the training group, 0.895, 95% CI (0.768-1.000) for the validation group, and 0.730, 95% CI (0.569–0.891) for the external test group. The nomogram model incorporating clinical data achieved superior results, the AUCs for predicting 2-year OS were 0.913 (95% CI: 0.83–0.98) for the training group, 0.86 (95% CI: 0.78–0.96) for the validation group, and 0.778 (95% CI: 0.69–0.94) for the external test group; for predicting 3-year OS, the AUCs were 0.837 (95% CI: 0.83–0.98) for the training group, 0.982 (95% CI: 0.84-1.0) for the validation group, and 0.785 (95% CI: 0.75–0.96) for the external test group. The calibration curve demonstrated excellent calibration of the model, while the decision curve and KM analysis indicated that the model possesses substantial clinical utility.

**Conclusion:**

The GBM model, based on the radiomic features of enhanced CT imaging, holds significant potential for predicting the prognosis of patients with muscle-invasive bladder cancer. Furthermore, the combined model, which incorporates clinical features, demonstrates enhanced performance and is beneficial for clinical decision-making.

**Supplementary Information:**

The online version contains supplementary material available at 10.1186/s12885-025-14279-6.

## Introduction

Bladder cancer ranks as the tenth most prevalent cancer and the thirteenth leading cause of cancer-related mortality globally [1, 2]. Muscle-Invasive Bladder Cancer (MIBC) constitutes approximately one-quarter of bladder cancer cases and is characterized as a highly aggressive malignancy of the urinary system [2, 3]. Radical Cystectomy (RC) serves as the primary treatment for this condition [4, 5]. However, due to the significant heterogeneity of bladder cancer, postoperative recurrence and mortality rates remain elevated even after radical treatment [4, 6], profoundly impacting patients’ quality of life and survival prospects [7, 8]. Consequently, accurately predicting the prognostic risk for MIBC patients is essential for informing treatment decisions and developing effective follow-up strategies, ultimately enhancing the prognosis for these patients.

Radiomics is an emerging field of imaging data research that differs from traditional methods, which rely on physicians’ subjective diagnoses of imaging data. This innovative approach converts medical images into high-dimensional data, enabling objective, non-invasive, and quantitative descriptions of tumor heterogeneity. Consequently, it provides valuable information for precise disease treatment [9–11]. When combined with machine learning techniques, radiomics can analyze and process large-scale, high-throughput data, facilitating the construction of predictive models that enable non-invasive assessment and evaluation of patients during diagnosis and treatment, ultimately leading to more accurate therapeutic outcomes [11–15]. Previous studies have employed various machine learning algorithms, including Random Survival Forest (RSF) and Survival Support Vector Machine (SurvivalSVM), in radiomics modeling across a range of diseases [16–18]. However, the performance of these algorithms often varies depending on the disease and the specific research objectives. This study aims to utilize multiple machine learning algorithms for radiomics modeling to predict the prognostic survival risk of patients with muscle-invasive bladder cancer (MIBC). Additionally, it seeks to integrate radiomics features with clinical characteristics to develop a clinical prediction model with optimal application value.

### Patients

This study collected preoperative CT imaging from 107 patients diagnosed with muscle-invasive bladder cancer (MIBC) sourced from The Cancer Imaging Archive (TCIA), along with clinical data obtained from the TCGA Portal system. Following a thorough examination and screening process, cases were excluded if they had missing survival data, survival times of less than 30 days, lacked bladder CT images, did not include contrast-enhanced CT, or contained CT images of insufficient quality for analysis. Ultimately, the clinical data of 91 patients were included in this study for the establishment and evaluation of the predictive model. All imaging and clinical data were de-identified and utilized with the approval of the institutional review board of the TCIA sponsoring institution.

**Fig. 1:**
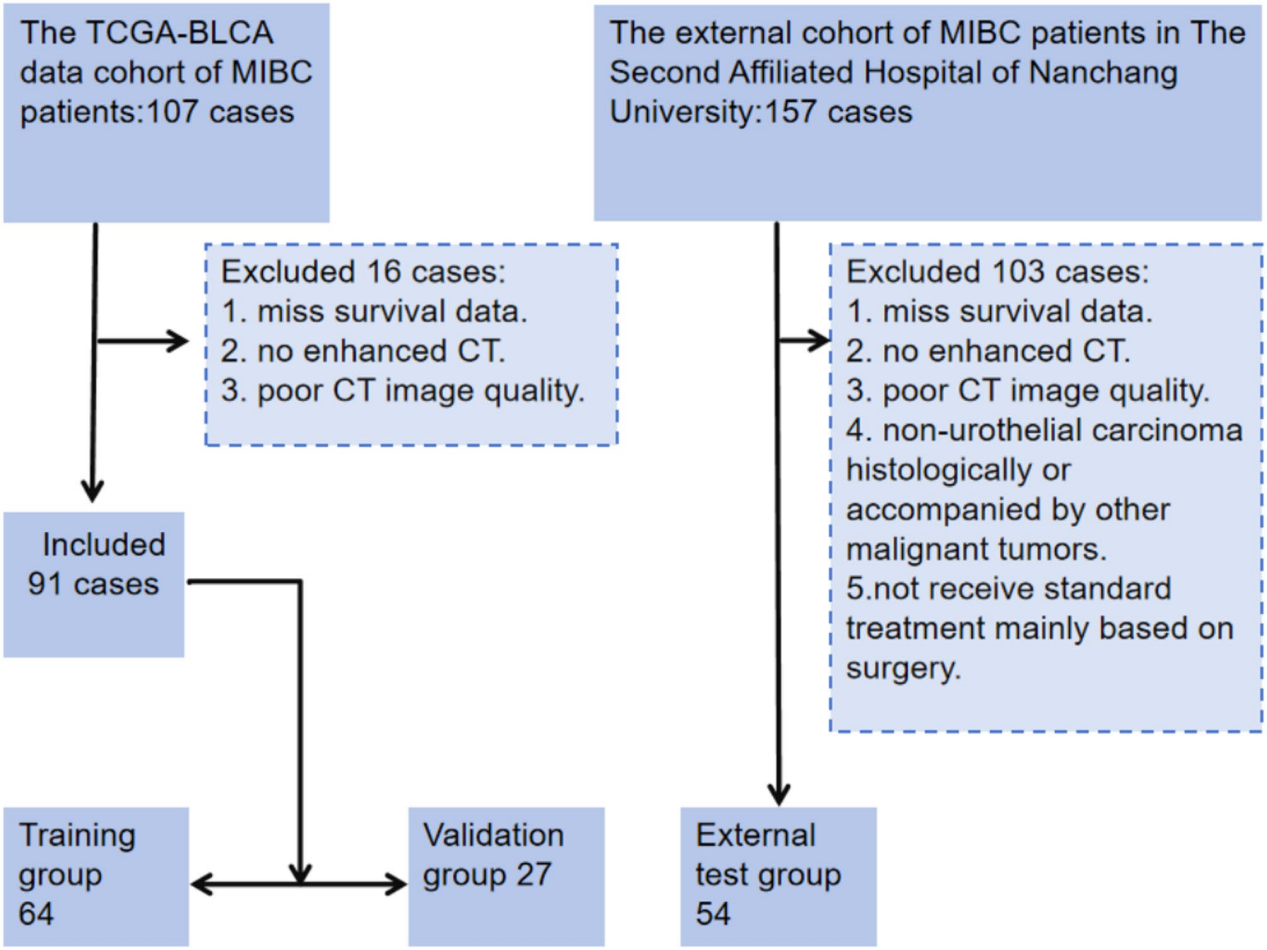
The flowchart of patient selection

The external validation cohort was retrospectively assembled from patients who underwent either partial or total surgical resection of the bladder at the Second Affiliated Hospital of Nanchang University between January 2018 and December 2020. Inclusion criteria encompassed: pathologically confirmed muscle-invasive bladder cancer (MIBC); either partial cystectomy or radical cystectomy; and the availability of a contrast-enhanced CT (Contrast-CT) scan conducted within two weeks prior to surgery. Exclusion criteria comprised: patients who received neoadjuvant chemotherapy; those with non-urothelial carcinoma confirmed by pathology; individuals with concurrent malignant tumors; and cases where CT images were either missing or of insufficient quality for analysis. A total of 157 patients were initially identified, but after screening, data from 54 patients were ultimately included in the study. The flowchart of patient selection was shown in Fig. 1. Clinical and pathological data for these patients were extracted from electronic medical records, encompassing age, gender, pathological TNM stage, and lymphovascular invasion present(LVI). This study adhered strictly to the ethical guidelines set forth in the Declaration of Helsinki and received approval from the hospital ethics committee (approval number to be added). Pathological tumor staging was conducted in accordance with the American Joint Committee on Cancer Staging Manual (8th edition).

### CT scanning plan for external validation cohort

Our hospital utilizes the Siemens Somatom Definition Flash dual-source CT machine for abdominal CT scanning. The scanning parameters are set to 120 kV, 210 mAs, with a slice thickness and spacing of 5.0 mm, and a matrix of 512 × 512. The reconstruction layer thickness is 1.25 mm. Initially, a routine plain scan is conducted, after which the contrast agent iopromide (370 mg I/mL) is injected through the cubital vein at a flow rate of 2.4 mL/s, administered at a dose of 1.1 mL/kg, followed by 20 mL of normal saline at the same flow rate to flush the tube. The arterial phase scan is delayed for 25 s, and the venous phase scan is performed 30 s after the initiation of the arterial phase.

### Image segmentation, delineation, and feature extraction

Two doctors from the urology department and two from the imaging department collaboratively reviewed the patients’ CT images. They utilized 3D Slicer (version 4.0) image processing software to accurately delineate bladder cancer. The region of interest (ROI) for bladder cancer (BC) was determined, with images of controversial cases decided upon after discussion. Given that the image data from the database and validation cohort were obtained using different equipment and acquisition conditions, all CT images were resampled to ensure a uniform voxel size of 1 mm × 1 mm × 1 mm, thereby eliminating resolution discrepancies between the various CT scanners. After this processing, both raw features and wavelet features were extracted from the delineated and processed CT image regions using the ‘pyradiomics’ toolkit in Python (version 3.7). Additional details regarding radiomics feature extraction can be found in the supplementary material.

### Feature data processing

Z-score normalization is applied to the extracted CT image feature data, wherein the value of each voxel is adjusted by subtracting the mean and dividing by the standard deviation. This process mitigates grayscale discrepancies across different CT scanners. Subsequently, the interquartile range method is employed to identify and remove outliers, with the outlier values being replaced by the mean to minimize their influence on the modeling. This study utilized univariate Cox regression analysis, least absolute shrinkage and selection operator (LASSO) Cox regression, and Spearman correlation analysis to assess the relationship between clinical characteristics and overall survival (OS). Radiation features were selected for modeling based on a P value of less than 0.05.

### Model construction and evaluation

**Fig. 2:**
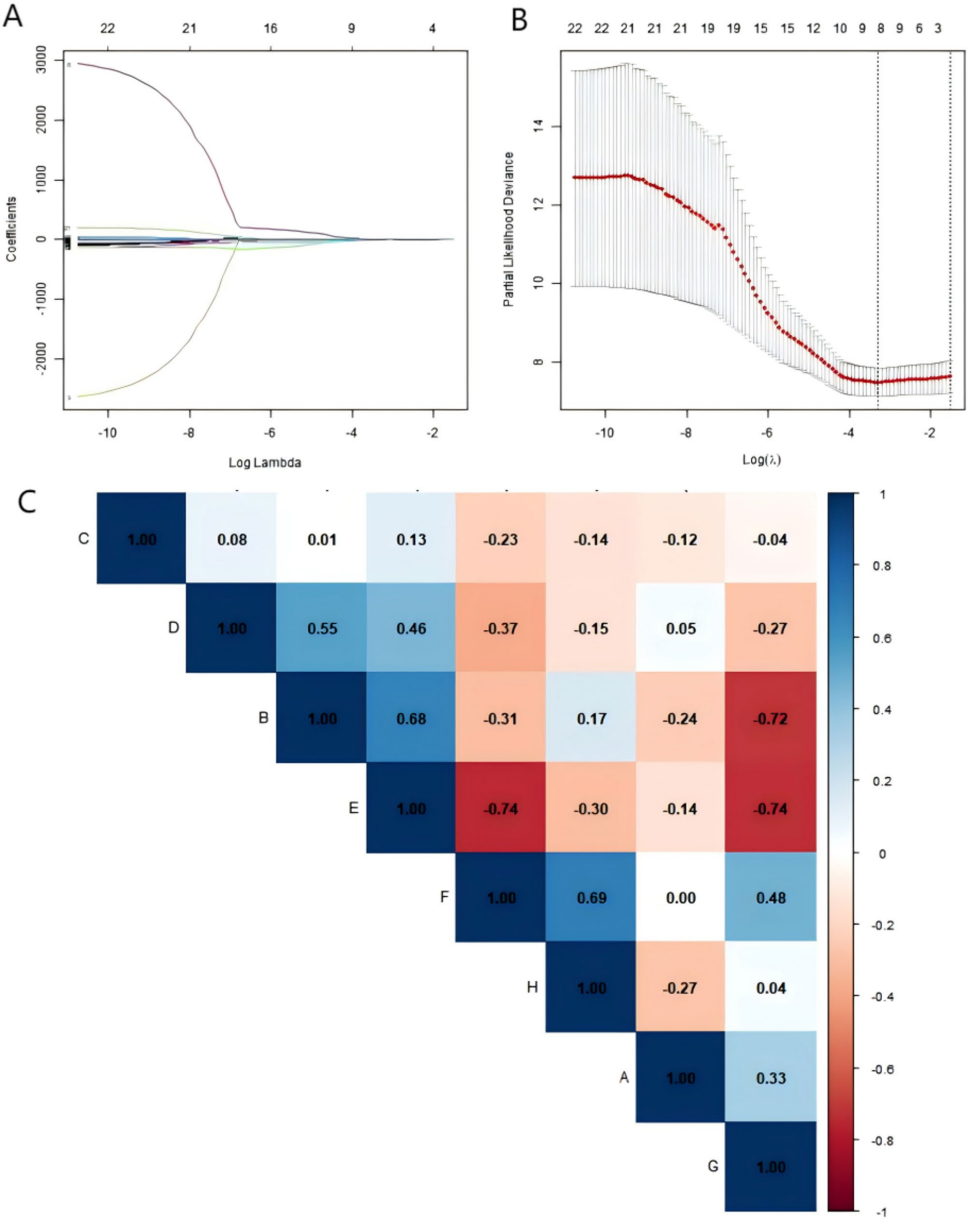
Lasso-Cox plot and Spearman heatmap for feature selection. (**A**) Path diagram of regression coefficients; (**B**) 10-fold cross-validation curve, with a vertical dashed line drawn at the lambda value corresponding to the minimum partial likelihood deviance; (**C**) Spearman correlation analysis heatmap

The TCIA database cohort was randomly divided into a training group and an internal testing group in a ratio of 7:3, while the cohort from our hospital served as the external validation group. Utilizing the optimal radiomic features identified through screening, five survival prediction machine learning algorithms were employed: gradient boosting machine (GBM), COXBOOT, COX, random survival forest (RF), and survival support vector machine (SSVM), to construct prognostic prediction models. These models were evaluated using 5-fold cross-validation, where applicable. Upon determining the optimal machine learning prediction model, it was designated as the radiomics score (rad-score) based on the model’s score. A joint nomogram was then constructed using significantly relevant clinical features. The overall workflow is depicted in Fig. 2. The discrimination of the model was verified using time-dependent AUC curves and ROC curves, while calibration curves were employed to assess the overall accuracy and calibration of the model. Additionally, clinical practice benefits were evaluated through decision curve analysis (DCA), and Kaplan-Meier survival analysis curves demonstrated the model’s capability to differentiate between high-risk and low-risk patient groups.

### Statistical analysis

Continuous variables are reported as medians with interquartile ranges (IQR), while categorical variables are expressed as frequencies and percentages. Baseline differences were assessed using the chi-square (χ²) test for categorical variables and one-way analysis of variance for continuous variables. Image data were extracted utilizing the ‘pyradiomics’ package in Python (version 3.7), and all other statistical analyses were conducted using R (version 4.4.2). P values less than 0.05 for all analyses were considered statistically significant.

## Results

### Baseline clinical characteristics of patients

Among the 91 patients in the entire TCGA cohort, 48% (44/91) died. The training group, divided at a ratio of 0.7, had 52% (33/63) patient deaths, while the validation group had a rate of 39% (11/28), and the external test group had 37% (20/54). A chi-square test was performed between the training group and the validation group, and the difference in rates between the groups was not statistically significant. The table lists detailed clinical patient characteristics for each subgroup. No statistically significant differences were found between the training group and the validation group in terms of age, T, N, and M staging, and lymphovascular invasion. Although there is a difference in the distribution of event counts between the external validation group and the TCGA group, the difference is not significant. To ensure the authenticity of the data, we did not employ data balancing techniques (See Table 1).

**Table 1:** Baseline clinical characteristics

characteristics	Train group(*n* = 63)	validation group(*n* = 28)	*p*	statistic	external test group(*n* = 54)	TCGA group (*n* = 91)	*p*	statistic
Event			0.354	0.858			0.185	1.76
Alive	30 (48)	17 (61)			34 (63)	47 (52)		
Dead	33 (52)	11 (39)			20 (37)	44 (48)		
age	67 (60, 76)	70.5 (64.75, 77.25)	0.355	990	72.5 (63.25,80)	68 (61, 76.5)	0.142	1.469
M stage			0.421	Fisher			< 0.001	-
M0	35 (56%)	13 (46%)			51 (94%)	48 (53%)		
Mx	26 (41%)	15 (54%)			0 (0%)	41 (45%)		
M1	2 (3%)	0 (0%)			3 (6%)	2 (2%)		
T stage			0.089	Fisher			0.364	2.019
T2	22 (35%)	4 (14%)			21 (39%)	26 (29%)		
T3	35 (56%)	19 (68%)			29 (54%)	54 (59%)		
T4	6 (10%)	5 (18%)			4 (7%)	11 (12%)		
N stage			0.196	Fisher			0.011	11.205
N0	31 (49)	19 (68)			39 (72%)	50 (55%)		
NX	9 (14%)	5 (18%)			9 (17%)	14 (15%)		
N1	10 (16%)	1 (4%)			6 (11%)	11 (12%)		
N2	13 (21%)	3 (11%)			0 (0%)	16 (18%)		
LVI			0.365	2.018			0.077	5.118
NO	17 (27%)	7 (25%)			18 (33%)	24 (26%)		
unknow	20 (32%)	13 (46%)			10 (19%)	33 (36%)		
YES	26 (41%)	8 (29%)			26 (48%)	34 (37%)		

T, N, and M represent pathological stages; LVI: status of lymphovascular invasion; *P* < 0.05 indicates statistical significance; data are assumed to be non-normally distributed by default, with continuous variables expressed as median (p25, p75) and categorical variables expressed as percentages

### Selection of features

A total of 836 image features were extracted from both the original conditions and wavelet transformations, Initially, 22 features significantly associated with the research objective were screened from the training group using univariate Cox regression analysis (P-value < 0.05), as detailed in Table 1 of the supplementary files. Subsequently, the Lasso-Cox regression method with 10-fold cross-validation was employed to further reduce the number of features. In the cross-validation results curve of the Lasso-Cox analysis (Fig. 2B), we identified the 8 most prognostically relevant radiomics features based on the minimum criterion (the lambda value with the lowest deviation) for further analysis, which are listed in Table 2 of the supplementary files. The Spearman correlation analysis (Fig. 2C) showed no significant correlation (> 0.8) among these 8 radiomics features.

### Models development and validation

**Fig. 3:**
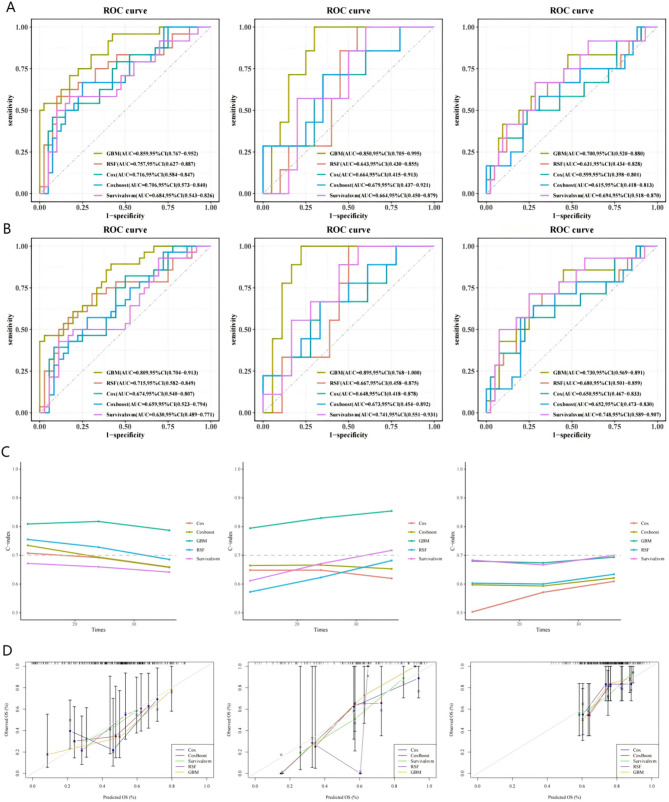
Evaluation results for 5 models. (**A**) ROC curves of the training group, validation group, and external test group at the 2-year time point; (**B**) ROC curves of the training group, validation group, and external test group at the 3-year time point; (**C**) C-index consistency index curves of the training group, validation group, and external test group at multiple time points; (**D**) Calibration curves comparing multiple models of the training group, validation group, and external test group at the 3-year time point

Based on the filtered radiomics features, we constructed five distinct machine learning models, with the hyperparameters of the models determined by the grid search method. The specific details of the hyperparameters can be found in Table 3 of the supplementary materials. In the evaluation results of five different models (Fig. 3), the overall performance of the Gradient Boosting Machine (GBM) is significantly superior to that of the other models. In the time-dependent C-index exponential curve (Fig. 3C), GBM demonstrated the highest consistency. The area under the curve (AUC) is an indicator reflecting the accuracy of predictions. For the GBM algorithm model, the 2-year AUCs were 0.859, 95% CI (0.767–0.952) for the training group, 0.850, 95% CI (0.705–0.995) for the validation group, and 0.700, 95% CI (0.520–0.880) for the external test group (Fig. 3A). The 3-year AUCs were 0.809, 95% CI (0.704–0.913) for the training group, 0.895, 95% CI (0.768-1.000) for the validation group, and 0.730, 95% CI (0.569–0.891) for the external test group (Fig. 3B). The calibration curve (Fig. 3D), obtained through 1,000 bootstrap self-sampling verifications, further substantiates the high degree of calibration exhibited by the GBM model. Additionally, while the survival support vector machine model (SurvivalSVM) does not achieve exceptional data learning and fitting performance, it maintains consistent performance across the training group, validation group, and external test group, reflecting good stability and versatility. Conversely, the classic Cox regression model demonstrates relatively low performance in ROC, AUC, C-index curves, and calibration curves.

### Model interpretability

**Fig. 4:**
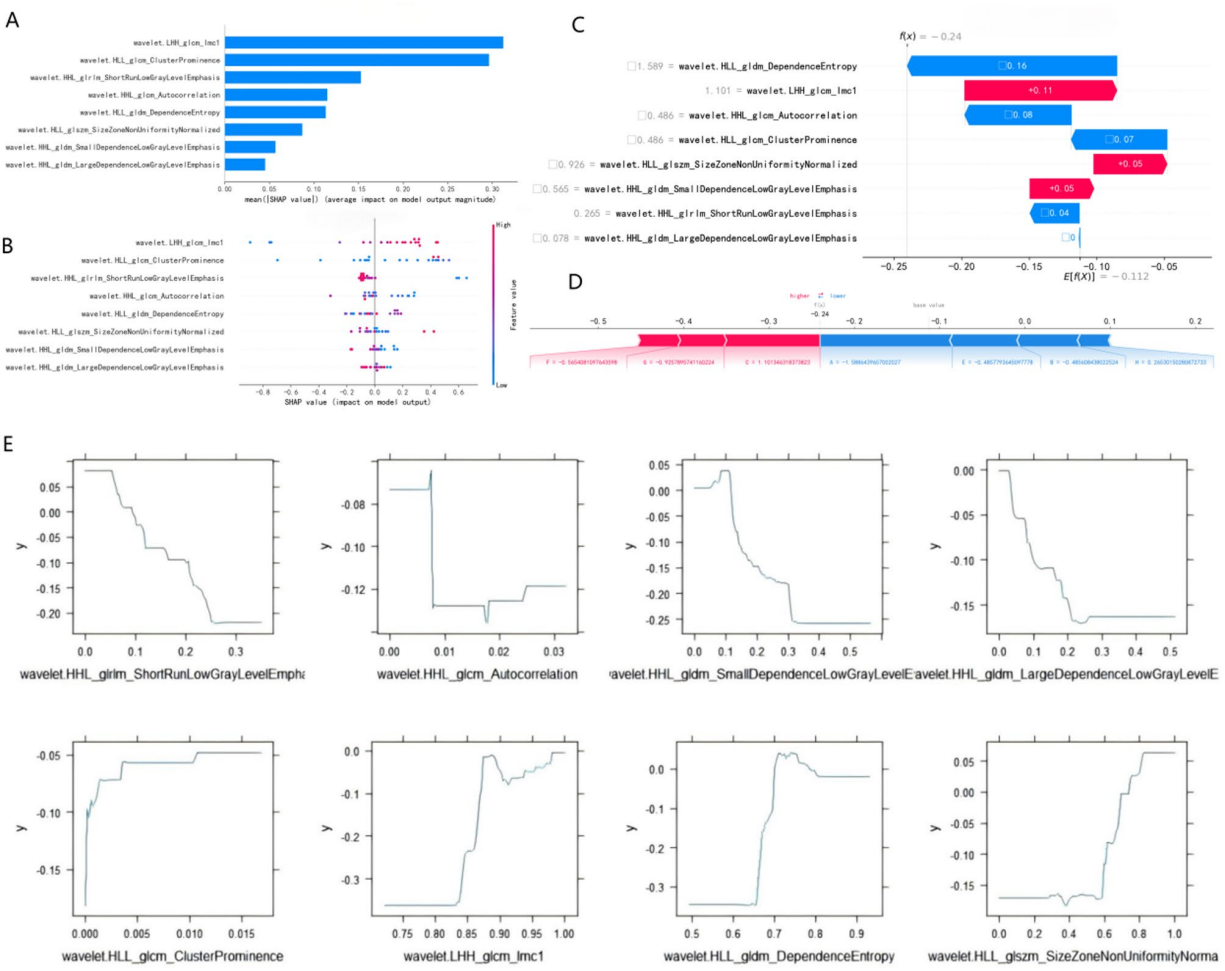
Machine learning interpretation of the GBM model based on SHAP (Shapley Additive Explanations). (**A**) SHAP bar chart displays the weights of the 8 most important features in the model. (**B**) SHAP swarm plot illustrates the positive or negative impact of each feature on the predicted probability through red and blue colors. (**C**) and (**D**) Examples of correctly predicted cases for the same low survival risk patient, (**C**) Waterfall plot of SHAP, (**D**) Force Plot, **A**: wavelet.HLL gldm DependenceEntropy, **B**: wavelet.HLL glcm ClusterProminence, **C**: wavelet.LHH glcm Imc1, **D**： wavelet.HHL gldm LargeDependenceLowGrayLevelEmphasis, **E**: wavelet.HHL glcm Autocorrelation, **F**: wavelet.HHL gldm SmallDependenceLowGrayLevelEmphasis, **G** wavelet.HLL glszm SizeZoneNonUniformityNormalized, **H**: wavelet.HHL glrlm ShortRunLowGrayLevelEmphasis.(**E**) Partial dependency plot of PDP for 8 features

We conducted SHAP interpretation and clinical application of the GBM model, calculating both global and individual Shapley values respectively. In the global visualization, the SHAP bar chart (Fig. 4A) displays the weight distribution of the model’s 8 features, and the SHAP swarm plot (Fig. 4B) illustrates the positive or negative impact of each feature on the predicted probability in red and blue. In predicting the probability of survival risk, three radiomics features—wavelet.LHH glcm Imc1, wavelet.HLL glcm ClusterProminence, and wavelet.HLL glcm DependenceEntropy—demonstrated positive impacts, while other features showed negative impacts.In the visualization of individual prediction results, Fig. 4C presents a typical example of correctly predicting low survival risk. The SHAP waterfall plot and force-directed graph (Force Plot) illustrate the positive or negative influence of each feature on the prediction outcome in individual cases. The base value (-0.112) represents the model’s fundamental prediction probability threshold, and f(x)=-0.24 indicates its individual final prediction probability.The Partial Dependence Plot (PDP) (Fig. 4E) illustrates the variation in the contribution of a single feature to the outcome variable when the data of other features remain unchanged. We can observe that changes in most radiomics features have a linear impact on the outcome. However, not all features follow this linear relationship. Features such as “wavelet.HLL_glszm_SizeZoneNonUniformityNormalized”, “wavelet.LHH_glcm_Imcl”, and “wavelet.HLL_gldm_DependenceEntropy” exhibit binary classification and more complex nonlinear characteristics in their association with outcomes. This finding may help explain why radiomics scoring systems “rad-score,” built upon simple linear associations in some studies, often fail to achieve desired predictive accuracy.

### Construction and evaluation of nomograms

**Fig. 5:**
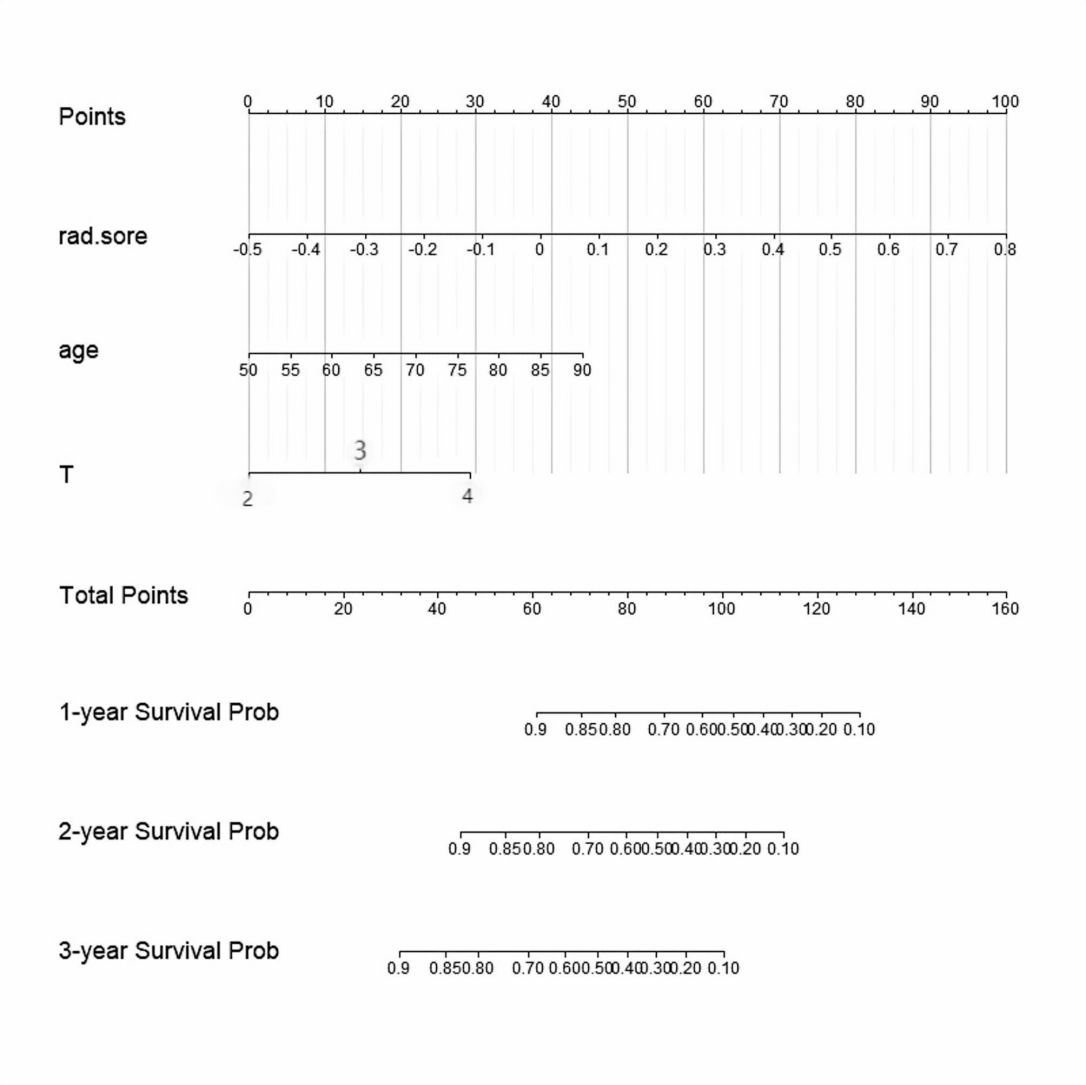
The nomogram developed from the training group data, integrating Rad-score, age, and pathological T stage

The results of univariate and multivariate COX regression analysis the training group showed that age and pathological T stage are independent risk factors for bladder cancer (Table 2). To utilize more predictive factors for better prediction performance and to make the model more user-friendly, we constructed a nomogram model by combining the radiomics model score (rad-score) with age and pathological T stage (Fig. 5). The rad-score in this model is directly defined by the risk score of the optimal model, the Gradient Boosting Machine (GBM) model, with specific values provided in Table 4 of the supplementary files.

**Table 2:** Results of univariate and multivariate COX analysis

characteristics	Uni-B	Uni-SE	Uni-HR	Uni-CI	Uni-Z	Uni-*P*	Multi-B	Multi-SE	Multi-HR	Multi-CI	Multi-Z	Multi-*P*
age	0.05	0.015	1.051	1.02–1.083	3.212	0.001	0.05	0.016	1.051	1.019–1.085	3.149	0.002
gender	-0.823	0.443	0.439	0.184–1.047	-1.857	0.063						
M	0.465	0.273	1.592	0.933–2.717	1.706	0.088						
T	0.562	0.255	1.755	1.065–2.893	2.206	0.027	0.556	0.266	1.744	1.036–2.937	2.092	0.036
N	0.133	0.124	1.142	0.896–1.456	1.071	0.284						
LVI	0.377	0.198	1.458	0.989–2.147	1.906	0.057						

B is the regression coefficient, SE is the Standard Error, HR is the Hazard Ratio, CI is the Confidence Interval, Z (Z-score) and P (P-value) are the results of the statistical significance test, and *P* > 0.05 represents statistical significance

**Fig. 6:**
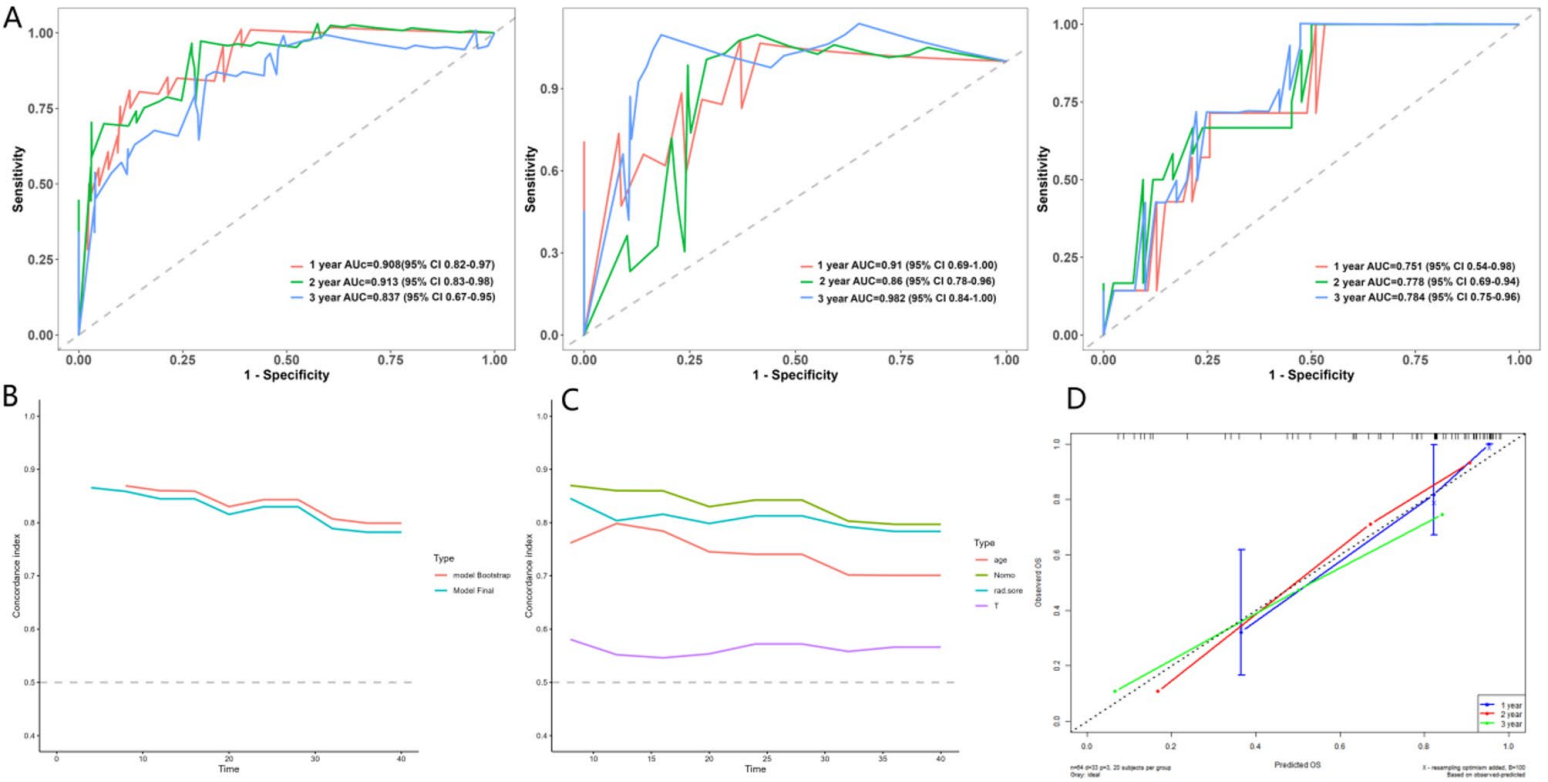
Validation of the nomogram. (**A**) ROC curves at multiple time points for the training group, validation group, and external test group; (**B**) Time-dependent AUC curves from bootstrap validation of the training group; (**C**) Multivariate time-dependent AUC curves of the training group; (**D**) Calibration curves of the training group

In the evaluation and validation of the nomogram, the AUCs for predicting 2-year OS were 0.913 (95% CI: 0.83–0.98) for the training group, 0.86 (95% CI: 0.78–0.96) for the validation group, and 0.778 (95% CI: 0.69–0.94) for the external test group; for predicting 3-year OS, the AUCs were 0.837 (95% CI: 0.83–0.98) for the training group, 0.982 (95% CI: 0.84-1.0) for the validation group, and 0.785 (95% CI: 0.75–0.96) for the external test group (Fig. 6A). The predictive discrimination is higher than models based solely on radiomic features. The calibration curve of the 200-time self-sampling bootstrap validation in the training group (Fig. 6D) demonstrates the excellent calibration of the model, while the time-dependent AUC curve plot from the 200-time self-sampling bootstrap validation illustrates the stability of the model (Fig. 6B). The multifactorial time-dependent AUC curve based on the training group data (Fig. 6C) shows that the predictive value of the rad-score is slightly higher than that of age, with pathological T staging being the lowest.

**Fig. 7:**
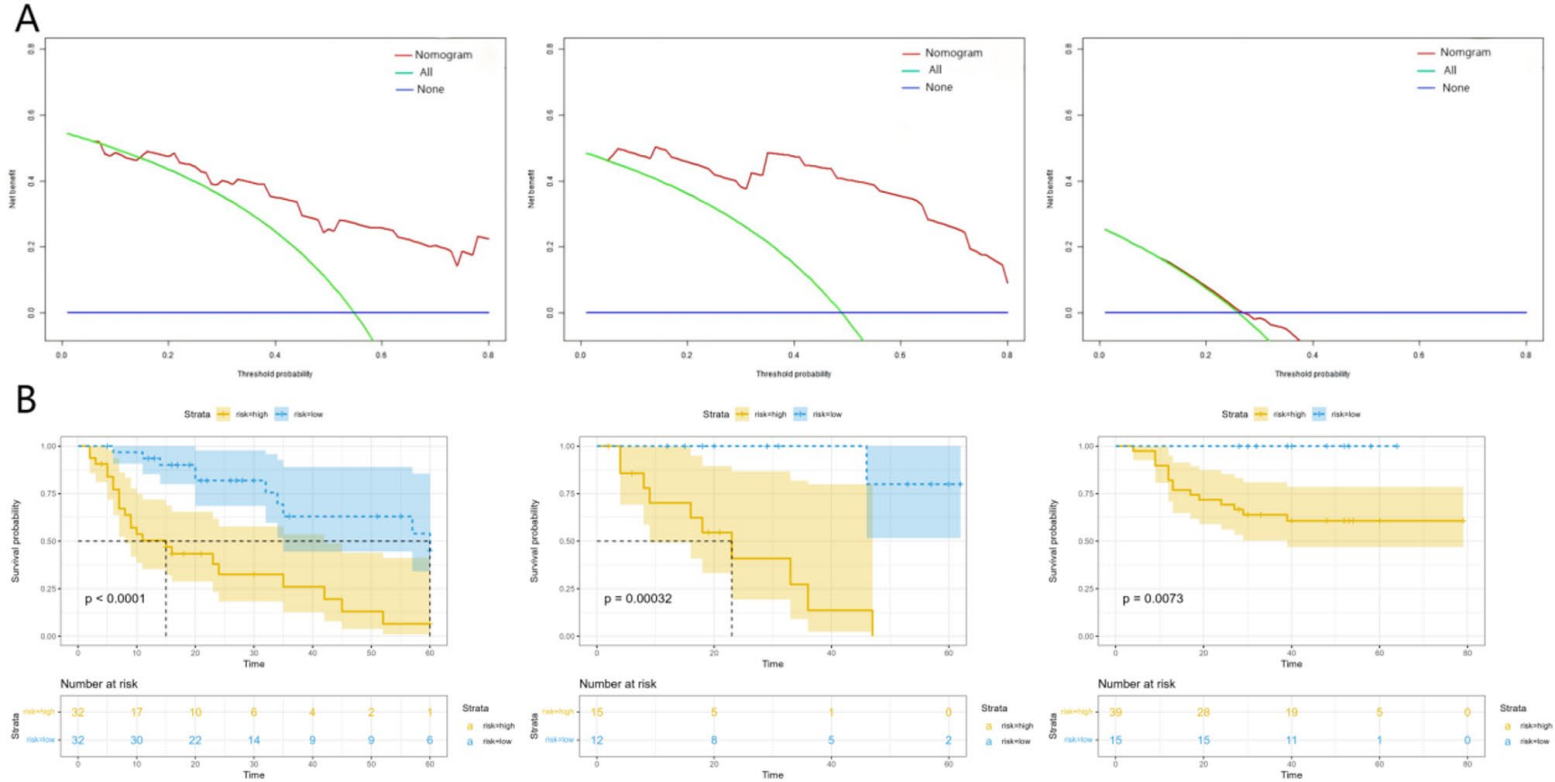
Decision curve (**A**) and KM curve (**B**) at the 3-year time point. (**A**) The decision curve, where “All” represents “intervene in all cases,” and “None” represents “intervene in none,” displays the threshold probability on the x-axis and the net benefit on the y-axis. A higher curve indicates a greater net benefit at that threshold probability. (**B**) The Kaplan-Meier curves based on the log-rank test sequentially show the differences in survival score stratification between the high-risk and low-risk groups in the training group, validation group, and external test group. The numbers of patients with high and low survival risks at different time points are displayed below the curves

The clinical utility and risk stratification capability of the nomogram model were evaluated. The decision curve analysis at the 3-year time point (Fig. 7A) demonstrated that the nomogram model could achieve high net benefits across all threshold probabilities in both the training and validation groups. However, in the external test group, the most optimal results were not achieved. At threshold probabilities exceeding 2.8, although the net benefit was higher than intervene in all cases, it was lower than intervene in none. The KM (Kaplan-Meier) curve at the 3-year time point (Fig. 7B) demonstrates that the nomogram score has excellent discriminative ability for stratifying patients’ survival risk, with P-values < 0.05 in the training set, test set, and external validation set. The OS of patients in the low-risk subgroup was significantly longer.

## Discussion

In this study, we utilized the TCGA-BLCA dataset from the TCGA database to conduct a radiomics modeling investigation aimed at predicting the prognostic risk of patients with muscle-invasive bladder cancer (MIBC), which was subsequently validated through a local external group. We developed multiple machine learning models and performed a comprehensive evaluation and comparison of their performance. The results indicate that the gradient boosting machine (GBM) exhibited the best overall performance among the models tested. Additionally, we constructed a nomogram model based on clinical data, which achieved the highest predictive accuracy. Given the poor prognosis associated with muscle-invasive bladder cancer, individualized diagnostic decisions and prognostic assessments are essential for patient management. Traditional assessment methods often rely solely on patients’ pathological results, neglecting the integration of other common clinical data, such as imaging information. Radiomics, as an emerging medical image analysis technology, can uncover both microscopic and macroscopic characteristics of tumors. In the diagnosis and prognosis prediction of many tumor diseases, there has been a relatively extensive body of research, particularly in liver tumors [19], breast tumors [20], and lung tumors [20]. Currently, clinical research in radiomics for bladder cancer primarily focuses on diagnostic prediction [21–26], while studies on survival prognosis remain relatively scarce.

In previous related studies, a multicenter validation study included 406 patients with muscle-invasive bladder cancer (MIBC) [27]. Based on CT imaging data and clinical information, the researchers established a deep learning (DL) score and a classical rad-score, respectively, and verified the advantages of deep learning through comparative analysis. Ultimately, the research team combined the deep learning score with clinical factors to construct a nomogram prediction model. However, this study has certain limitations. Firstly, the conventional deep learning method employed only performs convolution operations on two-dimensional images, which may lead to the loss of important three-dimensional structural information. Secondly, the comparison of the advantages of deep learning is based on rad-score, and rad-score is only established on a simple linear relationship, making it difficult to fully reflect the association between radiomic features and survival prognosis. Another earlier study, based on a single center, analyzed data from 301 patients who underwent radical cystectomy, employing the Cox regression model for training and validation, achieving a combined AUC value of 0.761 (95% CI: 0.617–0.874) for predicting overall survival [28]. Additionally, a study involving 163 patients utilized deep learning techniques combined with clinical data to construct a predictive model, which achieved an AUC performance of 0.87 ± 0.05 on the test group [29]. However, these previous studies were all confined to a single-center design, lacking external validation tests across regions and even national boundaries. Moreover, their modeling strategies were relatively singular, failing to sufficiently explore comparisons among various algorithms. Moreover, conventional deep learning methods carry the risk of overfitting due to insufficient sample sizes, and ordinary convolutional neural networks are unable to effectively extract three-dimensional volumetric information. Given that the actual number of cases for many clinical diseases is limited and the data variability is significant, traditional machine learning methods continue to demonstrate substantial application potential. Additionally, traditional machine learning methods possess strong interpretability. Although different machine learning methods have their respective strengths and weaknesses, we can compare the modeling results of various algorithms to select the algorithm model with the best fitting performance. This approach can be widely applied to various modeling requirements. Moreover, the association between radiomic features and survival prognosis may not be a simple linear relationship. The radiomics score (rad-score) constructed based on linear regression or classification models may have limitations, which is also one of the reasons why some prediction models solely based on this score perform poorly. The results of the PDP plot from our analysis and interpretation of the GBM model also support this point. Since machine learning can provide more complex and comprehensive data fitting capabilities, this study directly utilizes the score output of the optimal model, GBM, as the rad-score for the nomogram model, achieving better prediction results.

The TCGA and TCIA databases offer extensive cancer genome and imaging data, which can be utilized for various applications, including bioinformatics analysis, clinical correlation research, and the discovery of new drug targets [30]. Several radiomics studies have leveraged database-derived data to develop models for analyzing the prognosis of bladder cancer, identifying specific genes or gene products associated with patient outcomes. However, these findings have not yet undergone external validation [31–33]. Research methodologies that integrate public databases with local data not only enhance the model’s generalization capabilities but also validate its applicability across diverse populations and regions. Wang Xuanyi and Xie Tiansong, among others, utilized TCGA data alongside local patient cohorts and discovered that the nomogram model, constructed using radiomics scores, outperformed the TNM model in terms of disease-free survival (DFS) prediction. This model proved effective in predicting prognosis for patients with locally advanced breast cancer (LABC) following neoadjuvant chemotherapy and radiotherapy [34]. Similarly, Chen Siteng and colleagues developed a machine learning-based automatic diagnosis and prognosis model using pathomics features, leveraging the TCGA-BLCA training group and an external validation group from a local medical center. This model demonstrated strong performance in differentiating and predicting the prognosis of bladder cancer [35, 36]. However, it is noteworthy that radiomics studies combining the TCGA database with external validation remain relatively scarce.

Our results indicate that radiomics features are significantly associated with the overall survival (OS) of patients with muscle-invasive bladder cancer (MIBC) and can be utilized for prognostic assessment, aligning with findings from previous studies. Additionally, among the five machine learning models evaluated, the gradient boosting machine (GBM) demonstrated the most effective overall predictive performance. At the 3-year time point, the AUC values of the model were as follows: 0.809(95%CI: 0.704–0.913) for the training group; 0.895(95%CI: 0.768-1.000) for the validation group; and 0.730 (95%CI: 0.569–0.891) for the external test group. We employed the SHAP method to interpret the model. Additionally, by integrating the newly defined rad-score from the GBM model, pathological T stage, and patient age, a comprehensive nomogram was constructed, which demonstrated superior predictive performance compared to the standalone radiomics model. The AUC values of the comprehensive nomogram at the 3-year time point were as follows: training group 0.837 (95%CI: 0.83–0.98); validation group 0.982 (95%CI: 0.84-1.0); external test group 0.785 (95%CI: 0.75–0.96). The time-dependent AUC curve analysis of multiple factors revealed that the prognostic predictive value of Rad-score was significantly higher than that of pathological T stage, and slightly higher than that of age, which has been demonstrated by numerous studies as an important factor in patient survival prognosis [37, 38]. These results indicate that the machine learning prediction model based on radiomic features can achieve good prognostic prediction for muscle-invasive bladder cancer. The advantage of the nomogram lies in its visual tool presentation, which is easy to operate. The data relied upon by the model are all derived from routine clinical examinations and records, making data acquisition convenient. Moreover, the model can interface with the Electronic Health Record (EHR) system to automatically extract patients’ CT imaging data and clinical data, automatically invoke the nomogram model for prognostic evaluation through the system, and record the results in the patient’s medical record.

This study has the following limitations. First, there is heterogeneity in the data. Although all imaging data used to train the model are from the same database, these data were collected from various models of CT equipment in different medical institutions, and the sources of clinical information are diverse. Despite corresponding data processing, this heterogeneity may still affect the prediction results. Second, the number of patients included in the study is limited, which means that the generalizability of the survival risk assessment model still requires further verification and discussion. Additionally, due to the retrospective nature of data collection, there may be a certain degree of selection bias. The TCGA database and the local external test cohort contain partial clinical information that is not comprehensive, particularly in the specific records of adjuvant treatment regimens, where there are missing or difficult-to-standardize measurements. Therefore, when constructing the joint predictive model, these factors were not taken into consideration, although they actually have a significant impact on patient prognosis. In addition, immunohistochemical markers such as HER-2 [39] and Ki-67 [40], as well as indicators like the Systemic Inflammation Response Index (SIRI) [41] and Loss of Y Chromosome (LoY) [42], have been confirmed in previous studies to be closely related to the occurrence, progression, and other processes of bladder cancer, and they possess prognostic value for survival. Different methods of bladder resection and urinary diversion, as well as the patient’s mental and emotional state [43, 44], may all affect their prognosis. However, due to limitations in research conditions, we were unable to include more potential predictive factors. Finally, due to limitations in the hospital information system and individual patient circumstances, the follow-up period for the external test group was relatively short, with the majority of patients being tracked for only about three years. It is necessary to extend the observation period in the future to obtain more reliable data support. In summary, to further enhance the quality of the research findings, it is essential to continuously optimize and improve existing methods, expand the sample size, and incorporate data from more institutions for broader validation.

## Conclusion

This study successfully predicted the prognostic risk of MIBC patients through radiomics modeling, highlighting the pivotal role of this technology in the prognostic assessment of bladder cancer. This work not only deepens our understanding of MIBC prognosis but also provides new tools and technical means for the development of future personalized treatment plans. With the continuous advancement and maturation of the radiomics field, its application potential in the diagnosis and treatment of bladder cancer will become increasingly evident. Subsequent research should focus on enhancing the generalization performance of the models and validating their consistency and reliability through larger-scale, multi-center clinical trials.

## Electronic supplementary material

Below is the link to the electronic supplementary material.

Supplementary Material 1

Supplementary Material 2

Supplementary Material 3

Supplementary Material 4

## Data Availability

Partial CT image data and corresponding clinical information can be obtained from the TCGA (https://www.cancer.gov/tcga/) and the TCIA (https://www.cancerimagingarchive.net/) databases. The dataset of patients from our hospital involved in the study is not publicly available due to privacy regulations concerning patient information in our country. but it can be obtained from the corresponding author, Tao Zeng, upon reasonable request.

## Abbreviations

MIBC: Muscle Invasive Bladder Cancer
SVM: Support Vector Machine
RSF: Random Survival Forest
GBM: Gradient boosting machine
TCGA: The Cancer Genome Atlas
TCIA: The Cancer Imaging Archive
LASSO: Least absolute shrinkage and selection operator
ROC: Receiver operating characteristic
AUC: Area under curve
DCA: Decision curve analysis
PDP: The Partial Dependence Plot
SHAP: Shapley Additive explanations
